# Characteristics of psychosocial interventions to improve ART adherence in people living with HIV: A systematic review

**DOI:** 10.1371/journal.pgph.0000956

**Published:** 2022-10-26

**Authors:** Stefanella Costa-Cordella, Alejandra Rossi, Aitana Grasso-Cladera, Javiera Duarte, Claudia P. Cortes

**Affiliations:** 1 Centro de Estudios en Psicología Clínica y Psicoterapia (CEPPS), Facultad de Psicología, Universidad Diego Portales, Santiago, Chile; 2 Instituto Milenio Depresión y Personalidad (MIDAP), Santiago, Chile; 3 Centro de Estudios en Neurociencia Humana y Neuropsicología (CENHN), Facultad de Psicología, Universidad Diego Portales, Santiago, Chile; 4 Hospital Clínico San Borja Arriarán & Fundación Arriarán, Santiago, Chile; 5 Medicine Departament, Facultad de Medicina, Universidad de Chile, Santiago, Chile; University of California San Francisco, UNITED STATES

## Abstract

The HIV/AIDS pandemic continues to be a significant global public health crisis. The main HIV/AIDS treatment is the antiretroviral therapy (ART), which is highly effective but depends on the patient’s adherence to be successful. However, the adherence to antiretroviral therapy remains unsatisfactory across different populations, which raises considerable difficulties at both individual and collective levels. Suboptimal adherence to ART can be overcome through multidisciplinary management that includes evidence-based psychosocial interventions. Existing reviews on these interventions have focused mainly on studies with experimental designs, overlooking valuable interventions whose evidence comes from different study designs. Here, we aimed to carry out a comprehensive review of the current research on psychosocial interventions for ART adherence and their characteristics including studies with different designs. We conducted a systematic review following PRISMA guidelines. We searched five databases (Pubmed, EBSCO, LILACS, WoS and SCIELO) for articles reporting a psychosocial intervention to improve treatment adherence for people living with HIV (adults). The quality of each study was analyzed with standardized tools, and data were summarized using a narrative synthesis method. Twenty-three articles were identified for inclusion, and they demonstrated good to fair quality. Individual counseling was the most frequent intervention, followed by SMS reminders, education, and group support. Most interventions combined different strategies and self-efficacy was the most common underlying theoretical framework. This review provides insight into the main characteristics of current psychosocial interventions designed to improve ART treatment adherence.

**PROSPERO number:** CRD42021252449.

## Introduction

Human Immunodeficiency Virus (HIV) and Acquired Immune Deficiency Syndrome (AIDS) continue to be a significant global public health crisis [1–3]. According to the World Health Organization (WHO) and the United Nations Joint Programme on HIV/AIDS (UNADIS), 37.7 million people were living with HIV/AIDS (PLWHA) worldwide in 2020; 1.5 million people were newly infected, and approximately 680.000 people were dying of HIV/AIDS [4].

The treatment cornerstone of PLWHA is Antiretroviral therapy (ART). The effectiveness of ART therapy has been translated into a significant decrease in morbidity and mortality [5–8]. For ART to be successful, adherence to its prescribed dose is critical [9–12]. Optimal adherence, which is often considered to correspond to 95% or more of the prescribed doses taken [13], increases the effectiveness of the treatment, allowing an undetectable viral load to be reached [14]. ART operates by inhibiting virus replication, measured by viral load (i.e., the quantity of virus in the blood). When viral load is undetectable (i.e., below the detection level of the technique), the treatment is considered successful. Reaching undetectability has two benefits: the individual, which allows the patient’s immune system to recover, and the collective, which prevents the virus transmission and new infections. In contrast, suboptimal adherence to treatment favors the appearance of mutations in the virus, generating subtypes resistant to ART, which become predominant, enabling the transmission of resistant viruses in subsequent infections.

However, many patients have suboptimal adherence. A meta-analysis of 84 studies found that only 62% of patients took their prescribed doses at least 90% of the time [15]. It has also been found that adherence percentages vary between 27% and 80% across various cultural settings [16, 17].

Suboptimal adherence to ART is a multi-factorial and dynamic process directly associated with more health complications, mortality, and a higher risk of HIV transmission [18].

Causes of suboptimal adherence to ART vary between and within individuals, over time, and according to how adherence is defined or measured [16]. Although aspects of regimen complexity (e.g., daily dosing frequency, pill burden, intake requirements or prohibitions associated with food, and side effects) influence adherence [19], adherence to ART is most strongly associated with psychosocial factors [19, 20].

Several systematic reviews and meta-analyses have shown that the strongest predictors of treatment adherence are self-efficacy (i.e., a person’s belief in their capacity to perform the behaviors needed to reach a given goal), concerns about ART effectivity, trust/satisfaction with the HIV care provider, depression symptoms, HIV stigma, and social support [19, 21–24].

For this reason, non-pharmacological interventions that focus on psychological/social factors (i.e., psychosocial interventions) have been widely promoted as one of the best options to improve treatment adherence in people with HIV [25, 26].

In recent years, several systematic reviews and meta-analyses have investigated the effectiveness of psychosocial interventions in improving ART adherence [20, 27–35].

However, previous reviews focused mainly on the intervention’s outcomes and, thus, they only included studies with an experimental design, such as Randomized Controlled Trials (RCT). Nevertheless, as far as psychosocial interventions are concerned, multiple types of evidence (e.g., clinical observation, qualitative research, systematic case studies) are considered valid to nourish evidence-based practice, according to the American Psychological Association (APA).

For this reason, it might be fruitful to review the literature on interventions to improve ART adherence exhaustively. Thus, the general objective of the present study was to carry out a comprehensive review, including various research designs (i.e., randomized controlled trials, controlled intervention studies, case-control studies, before-after studies with no control group and qualitative studies) of the current research on psychosocial interventions for ART adherence and their characteristics. Specifically, in each of the selected papers, we aimed to identify the intervention’s: a) type; b) frequency and duration; c) Target group; d) underlying theory; c) supporting evidence and d) setting of delivery.

Therefore, we conducted a systematic review of published literature to show the diversity of interventions that aim to improve adherence to ART in PLWHA and describe the main characteristics of these studies to enrich the current perspective on different designs.

## Methods

We conducted a systematic review of the peer-reviewed academic literature. We registered the study protocol with the International Prospective Register of Systematic Reviews (PROSPERO; CRD42021252449). Additionally, the protocol and detailed methods are available in the Open Science Framework (osf.io/hctrf; 10.17605/OSF.IO/9MKRU). This review focused specifically on ART adherence. Other relevant concepts in HIV treatment were not included (e.g. retention in care, treatment adherence, self-efficacy, self-management) as this study is part of a larger project to improve ART adherence in Chile.

### Eligibility criteria

We included studies published in peer-reviewed journals at any time. Searches were conducted on 1 October 2021; the review itself was completed from 1 October 2021 to 1 November 2021.We aimed to include studies that presented a psychosocial intervention aiming to improve ART adherence in people living with HIV or AIDS. We understood psychosocial interventions as interventions using a social, psychological or behavioral approach, or a combination of these two, as defined by Laurenzi et al. [36]. The primary outcome for the studies included in this review was improvement in ART adherence assessed with any type of measure of ART adherence. Studies eligible for inclusion could include male or female adults and young adults living with HIV/AIDS as participants. We also included studies whose participants’ age ranges were below 18 years old if the average age was above 18 years old. As shown in Table 1, studies eligible for inclusion could include evaluations conducted as RCT, case studies, qualitative studies, pilot studies, protocols for RCT, prospective studies, pre-post studies and comparison trials. Studies with any comparison intervention (e.g., care as usual, other intervention, untreated) were included. There was no exclusion based on the primary comparator. We included all languages and geographical regions. More detailed information of inclusion exclusion criteria in Table 1.

**Table 1:** PICO, inclusion criteria, and exclusion criteria applied to literature search.

PICO	Inclusion Criteria	Exclusion Criteria
Population	PLWHA (Adolescents, Young Adults and Adults)	None
Intervention	Psychosocial Interventions	None
Comparison	None	None
Outcome	ART Treatment adherence/HIV treatment adherence when applicable	None
Study Design	Case Studies, RCT, Qualitative Studies, Pilot Studies, Protocols for RCT, Prospective Studies, Pre-Post Studies, Comparison Trials	None
Other	Published in peer-reviewed journals, any language, any geographical region, any year of publication	None

### Search method for identification of studies

Following PRISMA guidelines [37], we identified relevant studies by using a set of predetermined search terms (S1 Text) to systematically search numerous academic databases, including Pubmed, EBSCO, LILACS (Latinamerican and Caribbean Literature for Health Sciences), WoS (Web of Science) and SCIELO (see S1 Text for search strategy). The searches were developed and conducted by the investigators (S.C.C. & A.G.C.) without the collaboration of a librarian due to limited resources. All identified studies were exported to EndNote [38] where all duplicates were removed. After removing duplicates, we uploaded the titles and abstracts of the remaining articles into the collaborative spreadsheet software Google Sheets for screening [39].

Two reviewers (S.C.C and A.G.C) independently screened each article for eligibility by title and abstract. Cases of disagreement were discussed in a meeting between three authors (S.C.C, A.G.C and J.D) until reaching consensus.

### Data extraction

Three review authors (S.C.C, A.G.C and J.D) performed data extraction independently using the same online forms to codify: (i) Information about the studies (i.e., design, location, and results), and (ii) Information about the interventions (i.e., type of intervention, description, duration and frequency, target population, underlying theory, supporting evidence and setting). After independent review, data extraction was compared and disagreement resolved by consensus.

### Study quality and risk of bias assessment

Two researchers (S.C.C and A.G.C) independently conducted a quality assessment for each study using the NHBLI quality and risk of bias assessment Tool [40]. Each study was assessed with the design-pertinent scale (i.e., controlled intervention studies, case-control studies, before-after studies with no control group). In the case of RCT protocols, and pilot/feasibility studies we used the same scale as for full RCT, but we considered only the first five items, which were applicable before the implementation of the intervention. For qualitative studies, we used the quality assessment for the systematic review of qualitative evidence [41]. Disagreements between reviewers were resolved through discussion and consensus. (S1 Table).

### Synthesis method

We used a narrative synthesis method [42] to summarize the extracted data. A narrative synthesis method is a textual approach to the synthesis process and is widely used in systematic reviews, especially those focusing on broad questions beyond the interventions’ effectiveness [42]. Studies were grouped by intervention, population, outcomes and study design.

## Results

### Study selection

**Fig 1:**
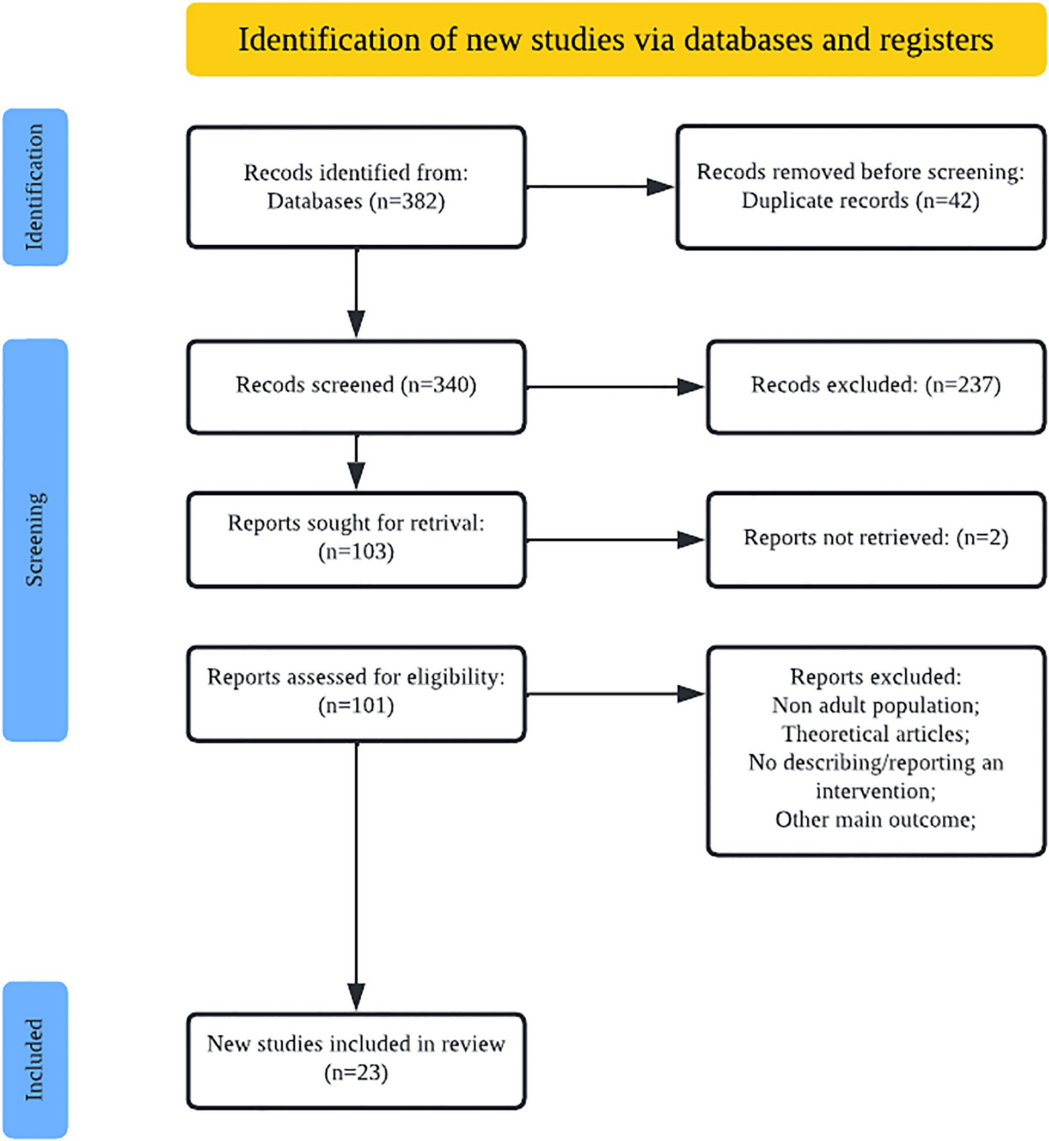
Identification of new studies via databases and registers.

Our initial search yielded 382 potentially eligible articles. After removing duplicates, the article titles were screened for inclusion/exclusion, and 237 articles were removed. The abstracts were reviewed, and eight more were excluded. The full texts of the remaining 93 were reviewed, and a further 70 were removed. As illustrated in Fig 1, a total of 23 studies met all of the inclusion criteria and were thus included in this review.

### Study characteristics

The studies reflected a variety of research designs. The most common design (n = 8) was protocols of Randomized Controlled Trial (RCT) followed by RCT (n = 6), pilot and feasibility studies (n = 5), qualitative studies (n = 2), and quasi-experimental (n = 2). The majority of the studies were conducted in North America (N = 11) or sub-Saharan Africa (N = 8). Only one study was conducted in Asia and two in Europe.

Regarding the findings, the majority of the RCT and quasi-experimental studies found a significantly bigger improvement in ART adherence in the intervention group [43–49]. Only in one RCT did both control and intervention groups improve their ART adherence levels, but with no significant differences [50]. Most pilot/feasibility studies were acceptable and feasible.

The main points of the reviewed studies are summarized in Table 2.

**Table 2:** Study characterization.

Reference	Design	Location	Results
*Kalichman et al*. *(2013)*	RCT	United States	Participants with moderated health literacy levels improved ART adherence but participants with low health literacy levels did not improve.
*Kalichman et al*. *(2016)*	RCT	United States	Counselling showed to be effective in enhancing ART adherence but there were no differences between groups with or without SMS.
*Bogart et al*. *(2017)*	RCT	United States	Larger effect on ART adherence over time in the experimental group. Importance of culturally tailored interventions.
*Dulli et al*. *(2020)*	RCT	Nigeria	ART adherence and retention in care improved significantly higher in the experimental group.
*Côté et al*. *(2020)*	RCT	Canada	ART adherence and self efficacy improved in both control and experimental groups.
*Attonito et al*. *(2020)*	RCT	United States	ART adherence and perceived social support levels improved in the Cognitive Behavior risk reduction group.
*Tanue et al*. *(2020)*	RCT Protocol	Cameroon	N/A
*Kim et al*. *(2020)*	RCT Protocol	Malawi	N/A
*Wagner et al*. *(2016)*	RCT Protocol	United States	N/A
*Orlando et al*. *(2021)*	RCT Protocol	Malawi	N/A
*Oberjé et al*. *(2013)*	RCT Protocol	Netherlands	N/A
*Duthely et al*. *(2020)*	RCT Protocol	United States	N/A
*Nsagha et al*. *(2020)*	RCT Protocol	Cameroon	N/A
*Bazzi et al*. *(2016)*	RCT Protocol	United States	N/A
*MacCarthy et al*. *(2020)*	Pilot/feasibility	Uganda	Acceptable and feasible
*Kalichman et al*, *(2019)*	Pilot/feasibility	South Africa	Feasible and potentially effective (ART adherence improved in experimental group)
*Rana et al*. *(2016)*	Pilot/feasibility	United States	Acceptable
*Been et al*. *(2020)*	Pilot/feasibility	Netherlands	Only peer support intervention was feasible.
*Kurth et al*. *(2016)*	Pilot/feasibility	United States	Acceptable and feasible
*Rodrigues et al*. *(2015)*	Qualitative	India	Perceived informational support from peer facilitators has greater impact and credibility than informational support from medical providers.
*Houston et al*. *(2015)*	Qualitative	United States	Both SMS and phone calls were perceived as signs of “care” from the clinic. Two-way communication was preferred to one-way. Caution with risk of unintentional disclosure of HIV status.
*Côté et al*. *(2015)*	Quasi-Experimental	Canada	ART adherence improved in both groups.
*Kunutsor et al*. *(2012)*	Quasi-Experimental	Uganda	ART adherence Improved in the group with enhanced pack vs care as usual.

RCT: Randomized Controlled Trial; Quasi-Experimental: clinical trials without randomization; PLWHA: People Living with HIV/AIDS;

### Quality assessment and risk of bias

Overall, the studies demonstrated good to fair quality. S1 Table provides details regarding the methodological quality assessment and overall risk of bias within and across studies for each outcome.

The majority of the reviewed studies (65%) were qualified as “good” (or “high quality” considering the terminology employed for qualitative studies) [43, 44, 47, 48, 50–62]. Five studies (21%) were qualified as “fair” [45, 46, 49, 63, 64], and only one (4.3%) was qualified as “poor” [65].

Among the good quality studies, five were RCT protocols [54, 56, 58, 62, 66], five were pilot and/or feasibility studies [51–53, 57, 61], four were RCT studies [43, 44, 47, 50] and one was a quasi-experimental study [48]. The “high quality” (qualitative) studies [59, 60].

Five studies qualified as “fair”. Of these, two were RCT [45, 46], two were RCT protocols [63, 64] and one was a quasi-experimental study [49].

**Fig 2:**
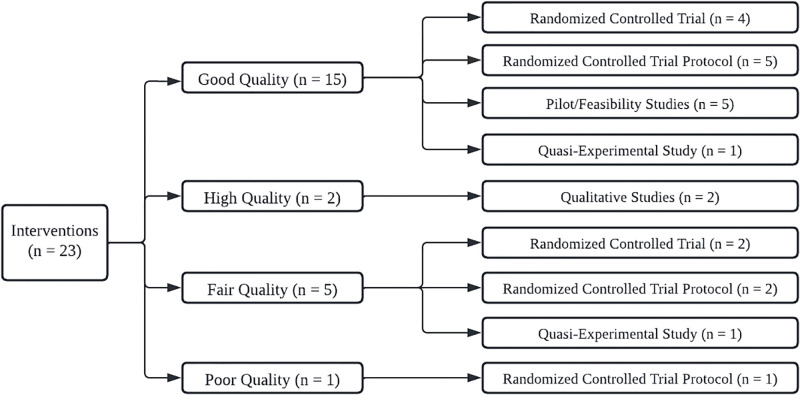
Characteristics and types of interventions analyzed.

The most common features within the fair-quality studies were the lack of validated measures to assess the study’s outcomes and the absence of blinding (although these criteria were not part of the assessment in the qualitative studies). The only research qualified as “poor quality” was an RCT protocol that did not report randomization techniques, blinding, and concealed treatment allocation. Fig 2 summarizes quality rating by research design.

### Intervention characteristics

#### Intervention type

Of the 23 interventions, 10 were counseling; three were educational interventions; six were SMS based interventions, and two were group support interventions. Two interventions were composed of a combination of interventions (both included counseling but not as the central intervention).

The main components of the interventions are described below (see also Table 3).

**Table 3:** Interventions characteristics.

Main author & year	Type	Main Components	Frequency and duration	Target Group	Underlying Theory	Evidence^1^	Setting
***RCT Studies***							
*Kalichman et al*. *(2013)*	Counselling	Counselling with educational components tailored specifically for people with limited health literacy.	3 sessions every 2 weeks	PLWHA and low literacy/health literacy	Self-efficacy/ Behavior Change	Yes	Community Organizations and Social Service Agencies
*Kalichman et al*. *(2016)*	Counselling and SMS	Phone delivered counselling with SMS reminders	5 sessions, bi-weekly (duration of intervention N/R)	PLWHA on ART and less than 95% adherence in the last month	Self-efficacy/ Behavior Change	Partly	Infectious Disease Clinics
*Bogart et al*. *(2017)*	Counselling	Culturally adapted counselling and identification of a person in the participant’s network who can support them in the treatment.	6 sessions during 5 months (weeks 1, 2, 4, 12 and 20)	Black/African American PLWHA on antiretroviral treatment	Ecological Model	Yes	Community-based AIDS Service
*Dulli et al*. *(2020)*	Group Support	Social Media Groups moderated by a trained adult LWHA. Discussions, educational material, games	Daily activities during 22 weeks	Young PLWHA on ART for less than 12 months	N/R	Yes	Primary Health Centers and Community Organizations
*Côté et al*. *(2020)*	Educational intervention	Web Based interactive sessions at a computer hosted by a virtual nurse who engages the user in a self-management skill-learning process. The platform adapts the content and messages to each user’s profile and needs.	4 sessions, 1-week interval	PLWHA on ART for at least 6 months	Self-efficacy/Behavior Change	Yes	Public Hospital
*Attonito et al*. *(2020)*	Group Support	Group level risk reduction intervention for an HIV infected drug using population.	8 sessions, 1 to 2 times per week	PLWHA and alcohol abuse	Information-Motivation-Behavioural skills (IMB) and Social Support	Yes	Community Organizations
***RCT Protocols***							
*Tanue et al*. *(2020)*	SMS	SMS with health messages, motivational messages, reminders. 2 x week. They contain a phone number to call if needed.	N/R	PLWHA on ART for at least 1 month	Health Belief Model	N/R	Government HIV Center
*Kim et al*. *(2020)*	Counselling + Educational	Culturally tailored educational videos + 10 minutes of scripted counselling delivered by a research assistant	N/R	Pregnant woman HIV+ not currently on ART	IMB	N/R	Urban Health Facilities and Rural District Hospital
*Wagner et al*. *(2016)*	Counselling	Sessions focus on the pre-treatment period. Pill-taking training and readiness for treatment assessment.	6 sessions during 24 months (every 2, 8, 14 weeks)	PLWHA not currently on ART (plan to start or restart)	IMB	Yes	HIV Clinics
*Orlando et al*. *(2021)*	Combination of interventions	3 simultaneous interventions: 1) one day of clinic open only to men 2) male delivered counselling for men 3) incentive for women who attend the clinic with their male partner	1 day	Woman living with HIV/AIDS and their partners	Ecological Model	No	Primary Health Centers
*Oberjé et al*. *(2013)*	Counselling	Nurse delivered counselling with a detailed protocol focused on reasons for non adherence and goals setting. Counselor and client meet until the goals are accomplished.	3 sessions every 2 to 4 months for 14 months	PLWHA with suboptimal adherence for 2 months	Self-efficacy/Behavior Change	Yes	HIV Clinics
*Duthely et al*. *(2020)*	SMS	SMS as reminders and SMS with psychoeducational information	Weekly or daily (duration of intervention N/R)	Woman living with HIV/AIDS	Health Belief Model	Yes	Women’s HIV Clinic
*Nsagha et al*. *(2020)*	SMS	Two-way SMS reminders and SMS with psychoeducational information.	3 times a week over 6 months	PLWHA on ART at least for 1 month	Nudge Theory	Yes	Regional and District Hospitals
*Bazzi et al*. *(2016)*	Couple counselling	Two sessions of counselling with the person LWHA and their partner. The aim is to strengthen the relationship to enhance motivation with HIV treatment.	12 sessions during 2 years (every 2 weeks, 1 and 6 months)	Males living with HIV/AIDS and their male partner	Principles of Cognitive Behavioural Therapy	Yes	Not specified
***Pilot/feasibility Studies***							
*MacCarthy et al*. *(2020)*	SMS	SMS with messages informing about the participant’s own adherence levels and those of their peers.	9 months (number of sessions and frequency N/R)	Young PLWHA	Behavioural Economics	No	Not specified
*Kalichman et al*. *(2019)*	Counselling	Phone-delivered counselling that directly addresses sources of stigma and alcohol consumption. 5 weekly calls.	5 weekly sessions	PLWHA on cART (HIV unsuppressed)	Self-efficacy/ Behavior Change	Yes	Public Health Clinic
*Rana et al*. *(2016)*	SMS	Bi-directional SMS with appointment and medication reminders, supportive messages and assistance with problem- solving	Weekly for 6 months	PLWHA new on ART/re-engaging with medical care/risk for ART non-adherence	N/R	No	HIV Clinics
*Been et al*. *(2020)*	Combination of interventions	4 interventions: directly administered ART, group medical appointment, screening of psychological distress, peer support for migrants LWHA	N/R	PLWHA	N/R	N/R	HIV Treatment Centers
*Kurth et al*. *(2016)*	Counselling	Computer-based counselling program culturally and linguistically adapted for spanish speaking population	N/R	Latin PLWHA on antiretrovirals	Technology Acceptance Model	Yes	University Hospital
***Qualitative Studies***							
*Rodrigues et al*. *(2015)*	SMS and Phone calls	Weekly SMS reminders and weekly interactive voice response phone calls	N/R	PLWHA (treatment naïve)	Self-efficacy/Behavior Change	Yes	University Hospital
*Houston et al*. *(2015)*	Counselling	Peer delivered counselling	12 months of intervention (number of sessions and frequency N/R)	African- American PLWHA (2–19 years from diagnosis)	Social Support	No	Public Hospital
***Quasi-Experimental Studies***							
*Côté et al*. *(2015)*	Educational Intervention	Web Based interactive sessions at a computer, hosted by a virtual nurse who engages the user in a self-management skill-learning process. The platform adapts the content and messages to each user’s profile and needs.	4 ***sessions*** in 8 months (frequency N/R)	PLWHA on ART for the last 6 months	Self-efficacy/Behavior Change	Yes	University Hospital
*Kunutsor et al*. *(2012)*	Combination of interventions	Enhanced adherence package including counselling, group education, leaflets, late attendee tracing and adherence diaries.	N/R	PLWHA on ART for at least 3 months	N/R	Yes	Government Hospitals and Center

N/R: Not Reported

N/A: Not Applicable (i.e., not relevant for the study design)

^**1**^Supporting evidence refers to the presence or absence of previous evidence on the intervention (Randomized Controlled Trials, implementation in other languages, Cultural Sensibility Study, among others).

#### Intervention’s main components

*Counseling*. Among the interventions using counseling, two integrated an educational component [44, 63], one included SMS as reminders of ART taking [43], and one directly addressed alcohol consumption and stigma [52]. One counseling was exclusively focused on the pre- ART treatment period [66]. Two counseling interventions concentrated on the participant’s partners; one was a couple’s counseling [65], and another offered counseling for women’s male partners [58]. Two counseling interventions were peer-delivered [58, 60], and two were part of a combination of other interventions [49, 58]. Two counseling interventions were delivered by phone [43, 52], and one was delivered by a virtual computer-based counselor [51].

*SMS*. Across SMS interventions, three consisted of health-related motivational messages and reminders [56, 59, 61]. Of these, one had bi-directional messages (i.e., the participant had the option to ask and receive an answer), and one had, in addition, bi-directional interactive voice calls. Two included psychoeducational messages [62, 64], and one included information regarding the participant’s adherence levels (as measured with pillbox technology) and their peers’ participants’ adherence levels [53].

*Educational*. There were three educational interventions; two of them used a platform with a virtual nurse guiding the users through client-centered information material [48, 50] and one used culturally adapted informational videos combined with counseling sessions [63].

*Group support*. Among group support interventions, there were two interventions delivered in a group format [46, 47]. One of them [47] consisted of an 8-sessions manualized group therapy led by two trained counselors, and they were focused on risk reduction in drugs using PLWHA. The other was a social media private group composed mainly of young PLWHA and led by an adult LWHA [46].

#### Intervention’s frequency

Counseling interventions duration was commonly between 3 and 6 sessions. Group support interventions were either weekly [47] or daily [46]. The interventions based on educational components were usually weekly sessions [50]. And the SMS interventions varied being weekly [43, 61, 62, 64] and daily [64]. Some studies did not report the frequency of the interventions [49, 51, 56, 57, 59, 63].

#### Intervention’s target groups

Ten of the reported interventions included patients with at least one month of ART treatment [43, 45, 46, 48–51, 56, 62, 67]. Some interventions were exclusively for women [58, 63, 64] and only one exclusively for men [65]. Two interventions had only HIV diagnosis as inclusion criteria [53, 57]. The remaining studies indicated more specific inclusion criteria such as being treatment-naive [55, 59], presenting low levels of treatment adherence [43, 54], substance abuse [47] and low health literacy [44].

#### Intervention’s underlying theory

The most common theoretical framework was the self-efficacy/Behavior Change [43, 44, 52, 68] which was used in four counseling interventions [43, 44, 52, 54], in two educational [48, 50], and one SMS [59].

The Information-Motivation-Behavioral (IMB) skills model was also a common underlying theory, and it was used in two counseling interventions [63, 66] and one group support [47], which was also framed considering social support theories. Social support theory was also the theoretical base of a peer-delivered counseling intervention [47, 60].

The Social-Ecological Model was used in two interventions: one counseling [45, 58] and one facility-based intervention that included peer counseling sessions [45, 58]. The health belief model was the theoretical framework of two SMS based interventions [56, 64], while the other similar SMS interventions used the Nudge Theory [62]. Behavioral economics is the underlying theory in the intervention using SMS with information about the participant’s levels of adherence and that of their peers [53]. Principles of Cognitive behavioral Therapy were the basis of the couples counseling intervention [65]. The Technology Acceptance Model was used in the intervention employing a computer-based counselor [51].

Finally, four of the reviewed articles did not report the underlying theory of their interventions [46, 49, 57, 61].

#### Supporting evidence

We assessed the presence or absence of previous evidence for the intervention described in the reviewed studies. Previous evidence included former RCTs evaluating the same intervention in other contexts and developing and testing previous cultural adaptations. Of the total number of interventions assessed (n = 23), 18 presented supporting evidence [44–52, 54, 57, 59, 62–66], only one had provided partial evidence [43], and five did not present any evidence [53, 56, 58, 60, 61].

#### Settings

The most common setting in which the interventions were delivered were specialized HIV centers (N = 10) [43, 45, 49, 50, 54, 56, 57, 61, 64, 66], followed by public hospitals (N = 5) [48, 50, 51, 59, 60], and primary health centers (N = 3) [46, 58, 63]. Some interventions were delivered in community organizations (N = 4) [44, 46, 47, 63], social services agencies (N = 1) [44] or in a regional hospital (N = 1) [62]. Two studies did not report the context of intervention delivery [53, 65].

## Discussion

According to the American Psychological Association (APA), evidence-based practice is composed of multiple types of research evidence. However, most systematic reviews of psychosocial interventions only include experimental designs in their analysis, as they are considered the highest quality evidence in medical practice. To address this gap, the present study carried out a comprehensive review of all current evidence on psychosocial interventions for ART adherence, regardless of study design. We analyzed the studies characteristics and assessed their quality to complement the information provided by systematic reviews that only include studies with experimental designs such as RCTs (e.g., [20, 29, 30, 32]). The latter is essential since all available tools and resources are important when improving adherence to ART in PLWHA.

The first finding that stands out from our review is that despite RCTs being considered the strongest type of evidence (type 1 evidence), our quality assessment shows that diverse study designs are of good quality. Our results reveal that studies with RCT, RCT protocols, pilot/feasibility, qualitative and quasi-experimental design scored high in quality. These findings show that a high-quality score is not something reserved for RCTs. For example, all qualitative studies scored for high-quality evidence. These studies provide relevant information for understanding in-depth adherence of ART for PLWHA. Qualitative methods applied to intervention’s development allow researchers to access and reveal participants’ experiences, providing essential information about what works well and how. This type of study needs to be developed to understand better what aspects of the interventions, the context, participants, and delivery are essential for adherence. Therefore, qualitative study designs should be included when analyzing the available evidence-based psychosocial interventions for a given problem. In this sense, reviewing intervention types and characteristics might be beneficial, especially in psychosocial interventions, to weigh potentially effective interventions.

Another interesting finding is that most interventions were centered on individual counseling and SMS reminders, while only two of the twenty-three studies focused on group intervention. Focusing on the individual rather than the group seems contradictory to evidence showing that support groups are the best way to help PLWHA deal with the stigma and discrimination [69]. Likewise, group treatment has been shown to provide good or excellent evidence for different mental health disorders such as depression and anxiety [70], which highly correlate with symptoms suffered by PLWHA. Similarly, although evidence shows that peer support is a substantial intervention with several advantages [71–73], only six studies included peer-related components (e.g., peer delivered counseling, peer group, among others). Peer support is crucial since it helps patients open up and feel understood by someone who has undergone similar experiences. In addition, implementing this kind of intervention is highly cost-effective [74–76].

Both group and peer-support components have been shown to boost the effectiveness of interventions [30, 45, 54, 59, 70, 77]. Hence, combining these types of intervention might be something to investigate further, since participants often report that having a sense of intimacy and being in a tailor-made intervention is what makes counseling appealing [78–80].

Another point to consider is that studies lack a more deep consideration of the training of the facilitators. Only half of the studies mentioned that the facilitators received training; however, developing clear protocols and formal guidance might help standardize the delivery of the interventions. Likewise, evidence shows that receiving formal training encourages commitment in the intervention’s facilitators [81–83]. Hence, this might be a feature to consider when designing interventions to improve adherence in PLWHA.

Our systematic review presents important limitations. First, we focused specifically on ART adherence. Future studies should consider adding other relevant aspects of HIV (e.g. retention in care, treatment adherence, self-efficacy, self-management). Second, we included only interventions customized for adults. Future reviews might include broader age ranges as interventions for populations such as young people living with HIV are extremely important. Third, our search strategy lacked the collaboration of a librarian, which would have helped develop a more precise and exhaustive inspection of scientific databases

Overall, our study provides evidence that supports the idea that there is a diversity of interventions that aim to improve adherence to ART in PLWHA whose quality is not necessarily linked to one type of study design. This is relevant when thinking about different ways to assess effectiveness in adherence to treatment and to design studies that better represent the target population.

## Conclusions

This systematic review aimed to provide a comprehensive review of the evidence on psychosocial interventions for ART adherence and their characteristics. We included diverse types of study designs, assessing the quality of the studies, identifying different kinds of interventions, target populations, the health facility in which the intervention was delivered, and the intervention’s facilitator and their training.

This review emphasizes the diversity of research designs reporting and assessing interventions to improve ART adherence in PLWHA. This diversity of approaches may, in turn, enrich both clinical practice and future research programs.

This review highlighted several factors researchers should consider when planning further research into adherence to treatment in PLWHA. First, studies using qualitative methodologies should be considered since they have proven useful to deepen the understanding of participant behavior and, considering what this review shows, they scored highly in the quality assessment. Second, despite the evidence showing its effectiveness, the low number of group interventions indicates the need for additional work with this type of intervention. Third, further detail regarding the target population and the health facilities should be included when thinking about improving treatment adherence. Finally, training facilitators emerged as a common factor in these interventions; thus, training should be further investigated as a relevant factor.

While the body of work available in the literature emphasizes the problem of adherence to ART in PLWHA, it does not appear to have thoroughly examined the contribution of different types of research designs that report and assess interventions. Thus, a specific assessment of each type of study design is recommended.

## Supporting information

S1 Text Search string used on PUBMED. (DOCX)

S1 Table Assessment and risk of bias. (DOCX)
