# Supplementary material for: Characteristics of psychosocial interventions to improve ART adherence in people living with HIV: A systematic review
Source: PLOS Glob Public Health. 2022 Oct 26;2(10):e0000956. doi: 10.1371/journal.pgph.0000956 (PMC10021974; doi:10.1371/journal.pgph.0000956)
Supplement: S1 Text — (DOCX) [file pgph.0000956.s001.docx]

**Supporting Information**

**S1 Text. Search String used on PUBMED.**

("hiv"[MeSH Terms] OR "hiv"[All Fields] OR "people living with HIV or AIDS"[All Fields] OR ("acquired immunodeficiency syndrome"[MeSH Terms] OR ("acquired"[All Fields] AND "immunodeficiency"[All Fields] AND "syndrome"[All Fields]) OR "acquired immunodeficiency syndrome"[All Fields] OR "aids"[All Fields])) AND "Psychosocial interventions"[All Fields]

The string was adapted for each platform.
