# Supplementary material for: Characteristics of psychosocial interventions to improve ART adherence in people living with HIV: A systematic review
Source: PLOS Glob Public Health. 2022 Oct 26;2(10):e0000956. doi: 10.1371/journal.pgph.0000956 (PMC10021974; doi:10.1371/journal.pgph.0000956)
Supplement: S1 Table — (DOCX) [file pgph.0000956.s002.docx]

**S1 Table. Quality Assessment and Risk of Bias.**

| ***Overall Quality Assessment and Risk of Bias*** | | | | | | | | | | | | | | | |
| --- | --- | --- | --- | --- | --- | --- | --- | --- | --- | --- | --- | --- | --- | --- | --- |
|  | **Criteria** | | | | | | | | | | | | | |  |
| **Study** | **1** | **2** | **3** | **4** | **5** | **6** | **7** | **8** | **9** | **10** | **11** | **12** | **13** | **14** | **Quality Rating** |
| *Quality assessment using the NHLBI Quality Assessment Tool for Controlled Intervention Studies* | | | | | | | | | | | | | | | |
| *Kalichman et al. (2013)* | Yes | Yes | Yes | Yes | Yes | Yes | Yes | Yes | CD | CD | Yes | Yes | Yes | Yes | GOOD |
| *Kalichman et al. (2016)* | Yes | Yes | Yes | NA | Yes | CD | Yes | Yes | CD | CD | Yes | Yes | Yes | Yes | GOOD |
| *Bogart et al. (2017)* | Yes | Yes | Yes | Yes | Yes | Yes | Yes | Yes | Yes | NR | No | Yes | Yes | Yes | FAIR |
| *Dulli et al. (2020)* | Yes | Yes | Yes | No | No | No | Yes | Yes | CD | Yes | Yes | Yes | Yes | Yes | FAIR |
| *Côté et al. (2020)* | Yes | Yes | Yes | No | Yes | Yes | No | Yes | Yes | Yes | Yes | Yes | Yes | Yes | GOOD |
| *Attonito, Villalba, & Dévieux (2020)* | Yes | Yes | Yes | NR | NR | Yes | Yes | Yes | Yes | Yes | Yes | Yes | Yes | Yes | GOOD |
| *Quality assessment using the NHLBI Quality Assessment Tool for Controlled Intervention Studies (Adapted for RCT Protocols)* | | | | | | | | | | | | | | | |
| *Oberjé et al. (2013)* | Yes | Yes | Yes | Yes | CD |  |  |  |  |  |  |  |  |  | GOOD |
| *Wagner et al. (2016)* | Yes | Yes | Yes | NA | Yes |  |  |  |  |  |  |  |  |  | GOOD |
| *Bazzi et al. (2016)* | Yes | NR | NR | NR | NR |  |  |  |  |  |  |  |  |  | POOR |
| *Tanue, Nsagha, Theophile, & Assob (2020)* | Yes | Yes | Yes | No | Yes |  |  |  |  |  |  |  |  |  | GOOD |
| *Kim et al. (2020)* | Yes | Yes | Yes | No | No |  |  |  |  |  |  |  |  |  | FAIR |
| *Nsagha, Siysi, Ekobo, Egbe, & Kibu (2020)* | Yes | Yes | Yes | Yes | Yes |  |  |  |  |  |  |  |  |  | GOOD |
| *Duthely, Sanchez-Covarrubias, Mohamed, & Potter (2020)* | Yes | Yes | Yes | NR | NR |  |  |  |  |  |  |  |  |  | FAIR |
| *Orlando et al. (2021)* | Yes | Yes | Yes | Yes | CD |  |  |  |  |  |  |  |  |  | GOOD |
| *Quality assessment using the NHLBI Quality Assessment Tool for Controlled Intervention Studies (Adapted for Pilot and Feasibility Trials)* | | | | | | | | | | | | | | | |
| *Kurth et al. (2016)* | Yes | Yes | NR | NR | NR | Yes | Yes | Yes | Yes | Yes |  |  |  |  | GOOD |
| *Kalichman, Mathews, Banas, & Kalichman, (2019)* | Yes | Yes | Yes | Yes | Yes | Yes | Yes | Yes | Yes | Yes |  |  |  |  | GOOD |
| *MacCarthy et al. (2020)* | Yes | Yes | Yes | CD | CD | Yes | Yes | Yes | CD | CD |  |  |  |  | GOOD |
| *Been et al. (2020)* | Yes | Yes | Yes | Yes | Yes | Yes | Yes | CD | CD | Yes |  |  |  |  | GOOD |
| *Rana, van den Berg, Lamy & Beckwith (2016)* | Yes | Yes | Yes | Yes | Yes | Yes | Yes | Yes | Yes | Yes |  |  |  |  | GOOD |
| *Quality assessment using the NHLBI Quality Assessment Tool for Before-After Studies with No Control Group* | | | | | | | | | | | | | | | |
| *Kunutsor et al. (2012)* | Yes | Yes | Yes | Yes | Yes | Yes | No | NR | Yes | Yes | No | NA |  |  | FAIR |
| *Quality assessment using the NHLBI Quality Assessment Tool for Case-Control Studies* | | | | | | | | | | | | | | | |
| *Côté, Rouleau, Ramirez-Garcia, & Bourbonnais (2015)* | Yes | Yes | Yes | Yes | Yes | Yes | Yes | Yes | Yes | Yes | Yes | Yes |  |  | GOOD |
| *Quality assessment using the NHLBI Quality Assessment Tool for Systematic Review of Qualitative Evidence* | | | | | | | | | | | | | | | |
| *Rodrigues et al. (2015)* | GOOD | GOOD | GOOD | GOOD | GOOD | GOOD | GOOD | GOOD | GOOD |  |  |  |  |  | HIGH |
| *Houston, Osborn, Lyons, Masvawure, & Raja (2015)* | GOOD | GOOD | GOOD | GOOD | GOOD | GOOD | GOOD | GOOD | GOOD |  |  |  |  |  | HIGH |

CD, cannot determine; NA, not applicable; NR, not reported
